# Developmental pathways to autism in tuberous sclerosis complex: Evidence from a longitudinal cohort

**DOI:** 10.1002/epi.70431

**Published:** 2026-08-10

**Authors:** Fiona S. McEwen, Abigail K. Runicles, Holan Liang, Emma Woodhouse, Lisa Underwood, Elizabeth Shephard, Edward D. Barker, Fintan Sheerin, Juul W. Steenbruggen, Tuberous Sclerosis 2000 Study Group, John R. W. Yates, Charlotte Tye, Patrick F. Bolton

**Affiliations:** ^1^ Department of War Studies King's College London London UK; ^2^ Department of Child & Adolescent Psychiatry, Institute of Psychiatry, Psychology, & Neuroscience King's College London London UK; ^3^ Social, Genetic, and Developmental Psychiatry Centre, Institute of Psychiatry, Psychology, & Neuroscience King's College London London UK; ^4^ South London and Maudsley National Health Service Foundation Trust London UK; ^5^ Autism Research Centre, Department of Psychiatry University of Cambridge Cambridge UK; ^6^ North East London National Health Service Foundation Trust London UK; ^7^ Forensic and Neurodevelopmental Sciences, Institute of Psychiatry, Psychology, & Neuroscience King's College London London UK; ^8^ Centre of Methods and Policy Application in the Social Sciences Research Centre, Faculty of Arts and Education Waipapa Taumata Rau, University of Auckland Auckland New Zealand; ^9^ Tuberous Sclerosis Complex New Zealand Auckland New Zealand; ^10^ Department of Psychology, Institute of Psychiatry, Psychology, & Neuroscience King's College London London UK; ^11^ Department of Neuroradiology Oxford University Hospitals Oxford UK; ^12^ Department of Genomic Medicine University of Cambridge Cambridge UK

**Keywords:** autism spectrum disorder, epilepsy, infantile spasms, longitudinal, tuberous sclerosis complex

## Abstract

The association between autism spectrum disorder (hereafter referred to as autism) and tuberous sclerosis complex (TSC) is well established, yet the developmental pathways linking genetic mutation, cortical pathology, and epilepsy with autism remain unclear. The Tuberous Sclerosis 2000 Study recruited children newly diagnosed with TSC (*N* = 125). Data on mutation status, cortical tuber burden (magnetic resonance imaging/computed tomography), seizure history, and cognitive ability were collected. Approximately 10 years later, follow‐up assessments of autism, cognitive ability, and epilepsy were completed (*n* = 86). Almost 40% of participants met diagnostic criteria for autism, with an additional 42% showing elevated autistic traits. Structural equation modeling identified two indirect pathways linking *TSC1/TSC2* mutation with the autism factor score: one via higher cortical tuber burden and infantile spasms and one via spasms alone. Concurrent seizure severity and lower intelligence quotient scores were also associated with higher autism factor scores. These findings provide preliminary longitudinal evidence supporting developmental associations between genetic vulnerability, cortical pathology, and early severe epilepsy with later autism. This highlights the importance of further investigation of early epileptic activity and associated neurodevelopmental outcomes in TSC and may inform understanding of epilepsy‐related pathways associated with autism.

**Key points**

- Autism prevalence: 40% of children with TSC met autism criteria, and 42% showed elevated autistic traits.
- Mechanistic pathways: structural equation modeling established two indirect pathways from *TSC1/TSC2* mutations to autism: one via cortical tuber burden and infantile spasms and one via spasms alone.
- Clinical covariates: increased concurrent seizure severity and lower IQ scores significantly correlated with higher autism factor scores.
- Neurodevelopmental impact: results offer longitudinal evidence linking early genetic vulnerability, cortical pathology, and severe early life epilepsy to autism.

## 1 INTRODUCTION

Autism spectrum disorder (hereafter referred to as autism) is a heterogeneous neurodevelopmental condition affecting 1% of the general population.^1^ It is characterized by difficulties in social communication, together with restricted, repetitive behaviors.^1^ Although autism has a high heritability (64%–91%),^2^ its diverse genetic and neurobiological underpinnings complicate the identification of underlying developmental mechanisms. Single‐gene conditions such as tuberous sclerosis complex (TSC) provide a valuable model for identifying developmental pathways linking genes and neurodevelopmental outcomes. In TSC, autism is markedly more prevalent, with 30%–60% of individuals meeting diagnostic criteria and many others exhibiting elevated autistic traits.^3^ These features form part of broader TSC‐associated neuropsychiatric disorders, affecting up to 90% of individuals.^4^

These neurodevelopmental differences are underpinned by the biological mechanisms of TSC.^5^ TSC is a multisystem genetic disorder caused by pathogenic mutations in the *TSC1* or *TSC2* genes, resulting in dysregulated mechanistic target of rapamycin (mTOR) signaling and widespread effects on brain development.^6^ Dysregulated mTOR contributes to the formation of cortical tubers, subependymal nodules, and white matter abnormalities.^5^, ^6^ Epilepsy affects 80%–90% of individuals with TSC and typically begins in infancy; approximately half develop infantile spasms, a severe epileptic encephalopathy associated with poor developmental outcomes.^6^

Although structural brain abnormalities,^5^, ^6^ early epileptiform activity,^3^, ^5^ and mutation type have each been linked to autism in TSC,^3^ most studies have examined these factors separately, leaving the interplay among these mechanisms unclear. Prior analyses from this cohort identified indirect pathways linking mutation, tuber burden, and early life seizures to intellectual ability^7^ and attention‐deficit/hyperactivity disorder (ADHD) symptoms.^8^ However, analogous pathways to autism have not been evaluated. We therefore used structural equation modeling (SEM) to examine whether cortical tuber burden and early life epilepsy contribute to associations between *TSC1/TSC2* genotype and autism.

## 2 MATERIALS AND METHODS

The Tuberous Sclerosis 2000 Study (TS2000) is a UK population‐based, prospective study of the natural history of TSC. Ethical approval and written informed consent were obtained at each phase (REC ref: 00/7/061, Supplementary Section 1 in Appendix S1 and Yates et al.^9^). In phase 1 (2001–2005), 125 participants met diagnostic criteria for TSC and completed study assessments (49.6% male, median age at recruitment = 2.7 years, range = 4 weeks–18 years). Clinicians assessed participants across UK clinics, obtaining a full medical history and a physical examination.^9^ During phase 2 (2012–2015), participants completed assessments of autism (*n* = 86) and cognitive ability (*n* = 88; 45% male, median age = 12.5 years, range = 7.8–26.9 years, 91.9% of the sample aged <18 years).

The causal mutation was determined for 96 children (*TSC1*, *n* = 19; *TSC2*, *n* = 77; Supplementary Section 1 in Appendix S1, Table S1). Cranial computed tomography or magnetic resonance imaging was available for most cases and reviewed in phase 1 (*n* = 86). In phase 2, more recent (*n* = 41) and new (*n =* 23) scans were obtained (total *n =* 109). Scans were coded by trained clinicians, blinded to outcomes (Supplementary Section 2 in Appendix S1, Table S2). Interrater reliability was acceptable. Epilepsy history was obtained from interviews, seizure diaries, and medical records, and summarized using the Early Childhood Epilepsy Severity Scale.^10^ Factor analysis indicated a single underlying severity dimension comprising of number of seizure types, duration, frequency at most severe, number of antiepileptic drugs, and treatment response. Severity factor scores were calculated for year 1 (*n* = 120), year 2 (*n =* 120), and 3 months prior to phase 2 assessment (age = 7+ years, *n =* 94). Additional scores were derived for spasm severity (all features excluding number of seizure types) and other seizure severity (all features including seizure types other than spasms) in the first 2 years, plus a lifetime “other seizure severity” score used to dichotomize cases into low versus high severity (mean difference *d* = 2.46; Supplementary Section 3 in Appendix S1, Table S2, Figure S4–S8).

Autism was assessed in 86 participants during phase 2 using the Autism Diagnostic Interview‐Revised (ADI‐R) and the Autism Diagnostic Observation Schedule (ADOS‐2), administered according to age and language level, with appropriate module and algorithm selection for minimally verbal participants (Supplementary Sections 4 and 6 in Appendix S1, Table S6). Categorical ADI‐R/ADOS‐2 scores were used to provide a best estimate clinical diagnosis by experienced child and adolescent psychiatrists, and a latent autism factor score (from ADI‐R diagnostic and current behavior algorithms and ADOS‐2 comparison scores; Supplementary Section 4 in Appendix S1, Figure S9) was generated via confirmatory factor analysis as a continuous measure of autistic traits. Further details are provided in Supplementary Section 4, Figures S9–12, and Supplementary Section 6 in Appendix S1, Table S6.

Intellectual ability was assessed at both study phases. In phase 1, children completed the Mullen Scales of Early Learning (MSEL) or the Vineland Adaptive Behavior Scales (VABS). In phase 2, cognitive ability was assessed using the Wechsler Abbreviated Scale of Intelligence‐II (WASI‐II), the British Picture Vocabulary Scale, and/or VABS‐II. Following previous work,^7^ a best estimate intelligence quotient (IQ) was derived for 124 participants using the highest quality available measure in the following order of priority: WASI‐II, MSEL, VABS‐II, then VABS (Supplementary Section 5 in Appendix S1).

### 2.1 Statistical analysis

#### 2.1.1 Aim 1: Neurobiological and epilepsy correlates of autism

Preliminary analyses examined associations between the autism factor score, cortical tuber burden, epilepsy characteristics (including infantile spasms and nonspasm seizure severity), and IQ (Supplementary Sections 4 and 6 in Appendix S1, Tables S5 and S6).

#### 2.1.2 Aim 2: Pathways from TSC genotype to autism

SEM was performed in Mplus (version 7.31) using full‐information maximum likelihood and 1000‐sample bootstrapping to estimate indirect effects and construct 95% confidence intervals (CIs), thereby providing robustness to missing data and nonnormality. Additional details and variable definitions are in Supplementary Sections 6 and 7 in Appendix S1, Table S7.

## 3 RESULTS

Of the 125 children enrolled in phase 1, 86 completed autism assessments at follow‐up and were broadly representative of the baseline cohort across demographic and clinical characteristics (Supplementary Section 1 in Appendix S1). Cortical tuber data were available for 109 participants; the mean total count was 19.3 (SD = 14.4), highest in frontal regions and lower in temporal, parietal, and occipital lobes (Supplementary Section 2 in Appendix S1, Table S2). Epilepsy was highly prevalent (91%), and 52% of participants experienced infantile spasms, which had an earlier onset and frequently co‐occurred with other seizure types. Exploratory factor analyses supported modeling spasms and nonspasm seizure types as distinct severity dimensions across the first 2 years of life (Supplementary Section 3 in Appendix S1, Figures S4–S6).

Autism was common; 39.5% of participants met full ADI‐R and ADOS‐2 criteria for autism, and an additional 41.9% met criteria on one instrument or had subthreshold levels of autistic traits. Total tuber count was positively associated with the autism factor score (rho = .27, *p* = .017). However, the presence of tubers in specific lobes was not associated with autism when controlling for total tuber count (Section 4.6 in Appendix S1, Table S5). Earlier seizure onset was associated with greater epilepsy severity and lower IQ but was not significantly associated with autism factor score (rho = −.20, *p* > .05; Supplementary Section 6 in Appendix S1, Table S6). Several seizure features, including spasms, status epilepticus, and other seizure types, were individually associated with elevated autism factor scores (Supplementary Section 4.7 in Appendix S1, Figures S13–S16). However, after accounting for estimated IQ, only infantile spasms remained significantly associated with the autism factor score.

### 3.1 Structural equation models

Bivariate associations between variables were used to guide specification of the SEM (Supplementary Section 6 in Appendix S1). The SEM (Figure 1) showed no direct effect of mutation on autism factor score. Instead, *TSC2* (vs. *TSC1*) mutation was associated with autism factor score through association with cortical tuber burden and early epilepsy. *TSC2* predicted higher cortical tuber burden, which in turn predicted greater infantile spasm severity and, more weakly, other seizure types. IQ was modeled as a correlated factor because autism symptoms and cognitive ability are strongly associated in TSC and may reflect shared genetic and developmental pathways. Modeling autism and IQ as correlated outcomes allowed for both overlapping and distinct predictors and enabled us to examine whether associations between epilepsy severity and autism remained after accounting for cognitive ability (Supplementary Sections 5 and 6 in Appendix S1, Figure S18, and Table S6). After accounting for this overlap, only infantile spasms were associated with autism factor scores, whereas both spasm and nonspasm seizures were associated with IQ scores. Two significant indirect pathways linked mutation to autism factor scores: via increased tuber count and spasms (*β* = 2.08, 95% CI = .15–8.02) and via spasms alone (*β* = 2.98, 95% CI = .04–8.89). These pathways were similar in magnitude and converged on spasms, indicating that infantile spasms may represent the primary epilepsy‐related pathway associated with autism variation in TSC. For bivariate associations and full SEM specifications, including alternative models and fit indices, see Supplementary Sections 6–7 in Appendix S1, Tables S6 and S7.

**FIGURE 1:**
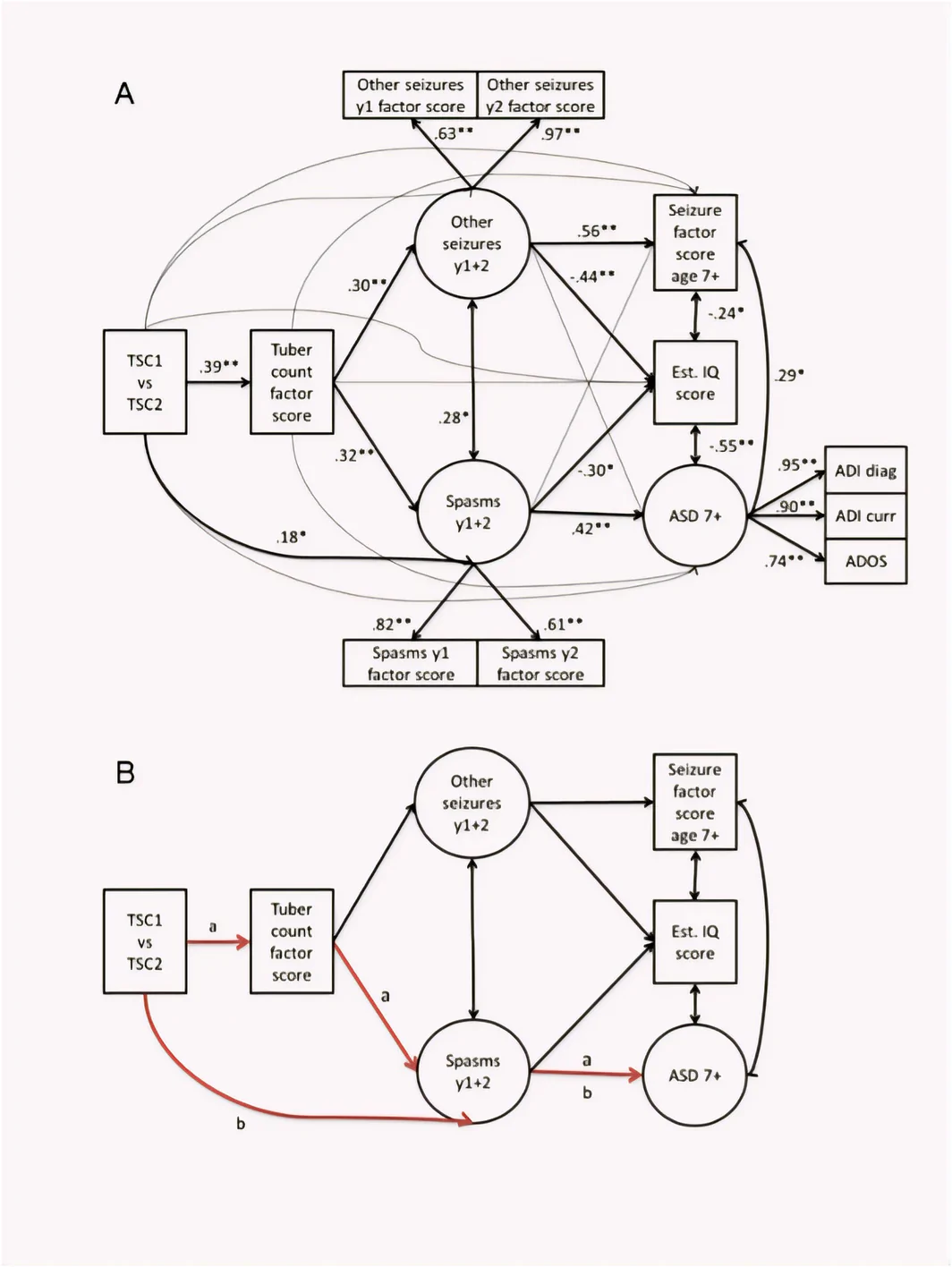
Structural equation model showing developmental pathways linking *TSC1*/*TSC2* mutation, cortical tuber burden, early life epilepsy, and autism factor scores. Black lines represent significant paths; gray lines represent nonsignificant paths. *TSC2* mutation was associated with higher cortical tuber burden, which in turn was associated with greater infantile spasm severity and, to a lesser extent, other seizure types in the first 2 years of life. Only spasms were associated with autism factor scores, whereas both spasm and nonspasm seizure dimensions were associated with intelligence quotient (IQ). Two significant indirect pathways linked mutation to autism: (1) mutation → tuber burden → spasm severity → autism (*β* = 2.08, 95% confidence interval [CI] = .15–8.02) and (2) mutation → spasm severity → autism (*β* = 2.98, 95% CI = .04–8.89). Model fit: *χ* ^2^_27_ = 37.43, p = .087; root mean square error of approximation = .056 (90% CI = .000–.095); comparative fit index = .974; standardized root mean square residual = .050. For full model specifications, comparison models, and additional fit indices, see Supplementary Section 7 in Appendix S1, Table S7. *p < .05, **p < .01. ADI, Autism Diagnostic Interview; ADOS, Autism Diagnostic Observation Schedule; ASD, autism spectrum disorder; curr, currently; diag, diagnosis; Est., estimated.

## 4 DISCUSSION

This study identified developmental associations between genotype, early neurological features, and autism in TSC. These findings extend previous TS2000 analyses demonstrating indirect effects of genotype on intellectual ability^7^ and ADHD symptoms,^8^ thereby supporting a developmental model in which genetic vulnerability, cortical organization and early epileptogenesis are associated with later behavioral outcomes. Earlier TS2000 analyses linked infantile spasms and epilepsy severity to later intellectual ability,^7^ suggesting shared developmental pathways between intellectual disability and autism. The present findings further implicate infantile spasms as an important epilepsy‐related pathway linking genotype to autism symptomatology.

Notably, the association between genotype and infantile spasms persisted after accounting for cortical tuber burden, suggesting contributions from both widespread cortical dysregulation and focal tuber pathology.^11^ Occurring during a critical period for the development of social and language networks,^12^ spasms may disrupt emerging connectivity patterns and contribute to both autism and intellectual impairment.^7^, ^13^ This finding aligns with previous studies identifying early epileptiform activity^3^, ^14^ and cortical abnormalities related to autism in TSC,^3^ as well as reviews emphasizing interactions among cortical pathology, network disruption, and mTOR pathway dysregulation.^5^

Clinically, the findings support further investigation of early epileptic activity and developmental outcomes in young children with TSC. Although vigabatrin is effective for seizure control^15^ and early treatment may improve short‐term outcomes,^16^ recent evidence suggests that presymptomatic therapy delays spasms but does not prevent autism or cognitive difficulties,^17^ consistent with intrinsic developmental vulnerabilities related to mTOR dysregulation. Emerging mTOR‐targeted therapies show promise; however, evidence for effects on behavioral outcomes remains limited.^18^

Limitations of this study include a modest sample size, which constrained model complexity and statistical power, and challenges in assessing autism in children with severe cognitive impairments, where overlap between autism‐related behaviors and cognitive difficulties may have influenced assessment scores. Future longitudinal studies incorporating neuroimaging, electrophysiology, and molecular biomarkers from infancy will be essential for identifying early markers of atypical development and informing targeted interventions.

## 5 CONCLUSIONS

In summary, autism factor scores in TSC were associated with developmental pathways linking *TSC2* mutation to cortical tuber burden and infantile spasms, with preliminary evidence supporting spasms as an important epilepsy‐related correlate of later autistic traits. Although early treatment may improve short‐term outcomes,^15^, ^16^ its relationship with long‐term autism risk requires further investigation.^18^ TSC therefore provides a useful model for understanding shared mechanisms underlying autism, epilepsy, and cognitive impairment.

## AUTHOR CONTRIBUTIONS

**Abigail K. Runicles:** Conceptualization; cowriting—original draft. **Fiona S. McEwen:** Conceptualization; data curation; formal analysis; investigation; methodology; project administration; supervision; validation; visualization; co‐writing—original draft. **Holan Liang:** Data curation; investigation; methodology; supervision; validation. **Emma Woodhouse, Lisa Underwood, Elizabeth Shephard:** Data curation; investigation; validation. **Edward D. Barker:** Formal analysis; methodology. **Fintan Sheerin:** Investigation; methodology. **Juul W. Steenbruggen:** Data curation; investigation. **John R. W. Yates:** Conceptualization; funding acquisition; methodology. **Charlotte Tye:** Conceptualization; data curation; investigation; methodology; supervision; validation; writing. **Patrick F. Bolton:** Conceptualization; funding acquisition; methodology; supervision; validation. All were part of the writing—review and editing and had approval of final draft.

## FUNDING INFORMATION

This study was supported by grants awarded to P.F.B. from Autism Speaks (grant no. 7696), the Baily Thomas Charitable Fund (grant no. Trust/RNA/AC/KW/36/4901), and the Tuberous Sclerosis Association (20122TS2000). C.T. was funded by a Medical Research Council studentship (G9817803) and a Tuberous Sclerosis Association Junior Fellowship. P.F.B. and C.T. were supported by the National Institute for Health Research (NIHR) Biomedical Research Centre at the South London and Maudsley NHS Foundation Trust and King's College London (P.F.B. by a NIHR senior investigator award, no. 2011). The views expressed are those of the authors and not necessarily those of the NHS, the NIHR, or the Department of Health and Social Care. The funders had no role in study design, collection, analysis, and interpretation of data, writing the report, or the decision to submit the article for publication.

## CONFLICT OF INTEREST STATEMENT

The authors declare no conflict of interest.

## ETHICS STATEMENT

Ethical approval for phase 1 was obtained through a Multicentre Research Ethics Committee and Local Research Ethics Committees for the participating centers (REC 00/7/061). Ethical approval for phase 2 was obtained through the NHS National Research Ethics Service, NHS Edgbaston (REC 00/7/061). The authors assert that all procedures contributing to this work complied with APA ethical standards in the treatment of humans, the ethical standards of the relevant national and institutional committees on human experimentation, and the Helsinki Declaration of 1975, as revised in 2008. We confirm that we have read the Journal's position on issues involved in ethical publication and affirm that this report is consistent with those guidelines.

## Supporting information


**Table S1.** Description of the sample by genetic mutation status.
**Figure S1.** Tuber load in whole brain (A) and each lobe separately (B–F).
**Figure S2.** Tuber load in whole brain shown by mutation status, for entire sample with tuber data (A; *N* = 109) and for subsample with both phase 2 autism assessment and tuber data (B; *n* = 80). NMI, no mutation identified; *TSC1,* mutation identified in *TSC1* gene; *TSC2,* mutation identified in *TSC2* gene.
**Table S2.** Cortical tuber count by major lobe and genetic mutation status.
**Figure S3.** Confirmatory factor analysis of cortical tuber load based on most recent or best quality scan.
**Table S3.** Exploratory factor analysis of seizure severity in the first 2 years of life.
**Figure S4.** Confirmatory factor analysis of seizure severity in the first 2 years of life. Factor loadings for two factors indicated by exploratory factor analysis: (1) seizure severity in year 1 and (2) seizure severity in year 2.
**Table S4.** Exploratory factor analysis of severity of spasm and other seizure types in the first 2 years of life.
**Figure S5.** Confirmatory factor analysis of epileptic spasms and other seizures in the first 2 years of life. Factor loadings for four factors indicated by exploratory factor analysis: (1) spasm severity in year 1, (2) spasm severity in year 2, (3) severity of other seizure types in year 1, and (4) severity of other seizure types in year 2.
**Figure S6.** Confirmatory factor analysis of seizure severity at 7+ years.
**Figure S7.** Boxplots showing distribution of (A) seizure severity factor score in year 1 and (B) epileptic spasms severity factor score in year 1 by mutation status. NMI, no mutation identified; *TSC1*, mutation identified in *TSC1* gene; *TSC2*, mutation identified in *TSC2* gene.
**Figure S8.** Boxplot showing distribution of age at seizure onset in months by mutation status. NMI, no mutation identified; *TSC1*, mutation identified in *TSC1* gene; *TSC2*, mutation identified in *TSC2* gene. Distributions are significantly different (Kruskal–Wallis test [*df*] = 14.20^3^, *p* = .003).
**Figure S9.** Confirmatory factor analysis of autism severity at 7+ years.
**Figure S10.** Autism spectrum disorder latent factor score in each diagnostic grouping.
**Figure S11.** Autism spectrum disorder (ASD) latent factor score in (A) all those with ASD data, (B) those who completed Autism Diagnostic Observation Schedule (ADOS‐2) Module 1 or 2 (nonverbal or minimally verbal), and (C) those who completed ADOS‐2 Module 3 or 4 (fluent speech).
**Figure S12.** Autism spectrum disorder factor score in those with (A) *TSC1* mutation (B), *TSC2* mutation (C), no mutation identified (D), and no genetic testing carried out.
**Table S5.** Correlations (Spearman rho) between tuber count and autism spectrum disorder (ASD) factor score (*n* with data in parentheses). ASD is factor score representing severity of symptoms. *p < .05, **p < .01.
**Figure S13.** Autism spectrum disorder factor score in those with (A) no history of epileptic spasms and (B) epileptic spasms.
**Figure S14.** Autism spectrum disorder factor score in those with (A) no history of status epilepticus and (B) at least one episode of status epilepticus.
**Figure S15.** Autism spectrum disorder factor score in those with (A) no history of epileptic spasms and no status epilepticus and (B) epileptic spasms and/or at least one episode of status epilepticus.
**Figure S16.** Autism spectrum disorder factor score in those with differing seizure history. No seizures, *n* = 11; status epilepticus (SE) only, *n* = 3; other seizure types only, *n* = 13; epileptic spasms only, *n* = 11; epileptic spasms and SE, *n* = 5; other seizure types and SE, *n* = 13; epileptic spasms and other seizure types, *n* = 14; epileptic spasms, other seizure types, and SE, *n* = 15.
**Figure S17.** Estimated marginal means for autism spectrum disorder factor scores by epilepsy risk factors.
**Figure S18.** Scatterplot of autism spectrum disorder (ASD) factor score by estimated intelligence quotient for each diagnostic group. No ASD, *R* ^2^ = .00; broad spectrum, *R* ^2^ = .33; probable ASD, *R* ^2^ = .20; ASD, *R* ^2^ = .17.
**Table S6.** Correlations (Spearman rho) between autism spectrum disorder (ASD) and all risk variables (*n* with data in parentheses). Spasms, other seizures, and ASD are all factor scores representing severity of symptoms. Only those with *TSC1* or *TSC2* mutation were included for correlations including genetic mutation status; those with no mutation identified or who were not tested were excluded. *p < .05, **p < .01.
**Table S7.** Fit statistics for structural equation models.
**Figure S19.** Structural equation modeling models tested.
**Appendix S1.** Supporting information sections.

## Data Availability

Readers seeking access to these data should contact Dr. Charlotte Tye, King's College London (charlotte.tye@kcl.ac.uk). Access to restricted and fully anonymized data will be granted to named individuals in accordance with research governance procedures governing the reuse of data. Specifically, requestors must complete a study data access request form with details of proposed data usage and a formal data sharing agreement. This may require additional ethical approval depending on the nature of the request. Code is available at https://osf.io/d2pk7/. All materials and methods are described and cited in the main article or supplementary materials. The study is reported in accordance with the STROBE Statement. This study utilizes data from a longitudinal study initiated in the year 2000 that was not preregistered, but the protocol is available on request.
