# Supplementary material for: Developmental pathways to autism in tuberous sclerosis complex: Evidence from a longitudinal cohort
Source: Epilepsia. 2026 Aug 10;67(10):e191–7. doi: 10.1002/epi.70431 (PMC13675775; doi:10.1002/epi.70431)
Supplement: Supplementary file 1 [file EPI-67-e191-s001.docx]

Supplementary Materials

Table of Contents

[**Supplementary Section 1. Description of sample**	3](#_Toc221007541)

[**Table S1. Description of the sample by genetic mutation status**	4](#_Toc221007542)

[**Supplementary Section 2: Tuber count**	6](#_Toc221007543)

[**Figure S1. Tuber load in whole brain (A) and each lobe separately (B-F)**	6](#_Toc221007544)

[**Figure S2. Tuber load in whole brain shown by mutation status, for entire sample with tuber data (A; N=109) and for subsample with both Phase 2 autism assessment and tuber data (B; N=80)**	7](#_Toc221007545)

[**Table S2. Cortical tuber count by major lobe and genetic mutation status**	7](#_Toc221007546)

[**Figure S3. Confirmatory factor analysis of cortical tuber load based on most recent or best quality scan**	8](#_Toc221007547)

[**Supplementary Section 3. Epilepsy**	9](#_Toc221007548)

[**3.1 Epileptic spasms**	9](#_Toc221007549)

[**3.2 Other seizure types**	9](#_Toc221007550)

[**3.3 Seizure history at Phase 2 assessment (current seizures)**	9](#_Toc221007551)

[**3.4 Factor analysis of seizure data**	9](#_Toc221007552)

[**3.5 Factor analysis of seizure severity in the first two years of life**	10](#_Toc221007553)

[**Table S3. Exploratory factor analysis of seizure severity in the first two years of life**	10](#_Toc221007554)

[**Figure S4. Confirmatory factor analysis of seizure severity in the first two years of life**	10](#_Toc221007555)

[**Table S4. Exploratory factor analysis of spasm and other seizure types severity in the first two years of life**	11](#_Toc221007556)

[**Figure S5. Confirmatory factor analysis of epileptic spasms and other seizures in the first two years of life**	11](#_Toc221007557)

[**3.6 Factor analysis of current seizure severity**	11](#_Toc221007558)

[**Figure S6. Confirmatory factor analysis of seizure severity at 7+ years**	12](#_Toc221007559)

[**3.7 Seizure severity and onset by genetic status**	12](#_Toc221007560)

[**Figure S7. Boxplots showing distribution of (A) seizure severity factor score in year 1 and (B) epileptic spasms severity factor score in year 1 by mutation status**	12](#_Toc221007561)

[**Figure S8. Boxplot showing distribution of age of seizure onset in months by mutation status**	12](#_Toc221007562)

[**Supplementary Section 4. Autism likelihood**	13](#_Toc221007563)

[**4.1 ADI-R algorithms**	13](#_Toc221007564)

[**4.2 ADOS-2 algorithms**	14](#_Toc221007565)

[**4.3 ASD research diagnosis**	14](#_Toc221007566)

[**4.4 ASD factor analysis**	15](#_Toc221007567)

[**Figure S9. Confirmatory factor analysis of autism severity at 7+ years**	15](#_Toc221007568)

[**Figure S10. ASD latent factor score in each diagnostic grouping**	15](#_Toc221007569)

[**4.5 ASD factor score distribution**	16](#_Toc221007570)

[**Figure S11. ASD latent factor score in (A) all those with ASD data, (B) those who completed ADOS-2 Module 1 or 2 (nonverbal or minimally verbal), and (C) those who completed ADOS-2 Module 3 or 4 (fluent speech)**	16](#_Toc221007571)

[**Figure S12. ASD factor score in those with (A) TSC1 mutation (B), TSC2 mutation (C), no mutation identified (D), and no genetic testing carried out**	17](#_Toc221007572)

[**4.6 Tuber location and ASD severity**	17](#_Toc221007573)

[**Table S5. Correlations (Spearman’s rho) between tuber count and ASD factor score (N with data in parentheses).**	18](#_Toc221007574)

[**4.7 Seizures and ASD severity**	19](#_Toc221007575)

[**Figure S13. ASD factor score in those with (A) no history of epileptic spasms and (B) epileptic spasms**	19](#_Toc221007576)

[**Figure S14. ASD factor score in those with (A) no history of status epilepticus and (B) at least one episode of status epilepticus**	20](#_Toc221007577)

[**Figure S15. ASD factor score in those with (A) no history of epileptic spasms and no status epilepticus and (B) epileptic spasms and/or at least one episode of status epilepticus**	20](#_Toc221007578)

[**Figure S16. ASD factor score in those with differing seizure history**	21](#_Toc221007579)

[**Figure S17. Estimated marginal means for ASD factor scores by epilepsy risk factors**	21](#_Toc221007580)

[**Supplementary Section 5. Intellectual ability**	22](#_Toc221007581)

[**5.1 Phase 1**	22](#_Toc221007582)

[**5.2 Phase 2**	22](#_Toc221007583)

[**5.3 Estimated IQ**	22](#_Toc221007584)

[**5.4 Estimated IQ and ASD**	22](#_Toc221007585)

[**Figure S18. Scatterplot of ASD factor score by estimated IQ for each diagnostic group**	23](#_Toc221007586)

[**Supplementary Section 6. Bivariate associations**	24](#_Toc221007587)

[**Table S6. Correlations (Spearman’s rho) between ASD and all risk variables (N with data in parentheses).**	25](#_Toc221007588)

[**Supplementary Section 7. Structural Equation Models (SEM) tested**	26](#_Toc221007589)

[**Table S7: Fit statistics for Structural Equation Models**	26](#_Toc221007590)

[**Figure S19. SEM models tested**	29](#_Toc221007591)

[**References**	30](#_Toc221007592)

# **Supplementary Section 1. Description of sample**

The Tuberous Sclerosis 2000 (TS 2000) Cohort Study

1. Phase 1 (2001-2005). Ethical approval for Phase 1 was through a Multicentre Research Ethics Committee and Local Research Ethics Committees for the participating centres (REC 00/7/061). Children aged 0–16 years resident in the UK with definite or possible TSC diagnosed between 1 January 2001 and 31 December 2005 were ascertained through paediatricians, paediatric neurologists and clinical geneticists and the UK Tuberous Sclerosis Association by mailing at the start of the recruitment period and annually thereafter. Current diagnostic criteria^1^ were used and cases with a possible diagnosis of TSC were included because young children with TSC do not always meet the criteria for a definite diagnosis when they first present. The final sample was N=125 (N=62 male).
2. Phase 2 (2012-2015). Ethical approval for Phase 2 was obtained through the NHS National Research Ethics Service, NHS Edgbaston (REC 00/7/061). This follow up took place approximately 10 years after Phase 1. A team of research psychologists re-contacted families and invited them to complete behavioural and cognitive assessments, either in the family’s home or at the research centre. Medical data were also collected, including recent seizure history, brain scan, and other data relevant to TSC where available. Several attempts were made to contact all families; where it was possible to contact families but they did not want their child to complete behavioural and cognitive assessments, consent was sought to collect data from parents over the phone (e.g., seizure data) and/or collect medical data from the child’s doctors. N=86 children completed Phase 2 assessments for ASD; the number of participants with other data are specified in the main manuscript.
   Children who completed ASD assessments were representative of the original sample in terms of gender (45.3% vs. 49.6% male, p=.248), mutation (TSC1 mutation, 18.6% vs. 15.2%, TSC2 mutation, 60.5% vs. 61.6%, no mutation identified, 4.7% vs. 5.6%, not tested, 16.3% vs. 17.6%; p=.826), total cortical tuber count (mean (SD), 18.88 (14.66) vs. 19.31 (14.42), p=.791), mean seizure severity (ever) rating at Phase 1 (mean (SD), 6.25 (4.42) vs. 6.28 (4.63), p=.958, history of epileptic spasms (percentage with spasms, 53.5% vs. 52.1%, p=.440), history of status epilepticus (38.0% vs.41.9%, p=.461), or estimated IQ at Phase 1 (mean (SD), 67.76 (21.14) vs. 67.43 (20.25), p=.888). Median age of seizure onset was slightly but significantly higher in those who completed autism assessments (6.00 vs. 5.00 months, p=.002). Thus other than this small difference, the sample who completed Phase 2 assessments were representative of the sample recruited during Phase 1.

### **Table S1. Description of the sample by genetic mutation status**

|  | N (%) of sample with data | Overall | TSC1 mutation (n=19) | TSC2 mutation (n=77) | No mutation identified (n=7) | Not tested (n=22) | χ^2^(df) or F(df) | Posthoc test |
| --- | --- | --- | --- | --- | --- | --- | --- | --- |
| Sex, M:F | 125 (100%) | 62:63 | 14:5 | 32:45 | 5:2 | 11:11 | 7.63 (3), p=.055 |  |
| Age of autism assessment in years;months, mean (SD) [range] | 86 (68.8%) | 13.1 (3.8) [7.8-26.9] | 13.8 (4.1) [9.3-25.8] | 12.7 (3.7) [7.8-26.9] | 11.6 (4.5) [8.0-17.8] | 14.1 (3.8) [9.9-24.6] | 0.94 (3,82), p=.423 |  |
| Cortical tuber count for all major lobes, mean (SD) [range] | 109 (87.2%) | 19.31 (14.42)  [0-68] | 6.13 (6.40) [0-22] | 22.01 (14.54) [0-68] | 27.86 (14.92) [7-48] | 16.89 (12.16)  [0-43] | 6.96 (3,105), p<.001 | TSC1 < TSC2 / NMI / no testing |
| Seizures ever, n (%) | 125 (100%) | 114 (91.2%) | 16 (84.2%) | 72 (93.5%) | 7 (100.0%) | 19 (86.4%) | 2.98 (3), p=.422 |  |
| Epileptic spasms ever, n (%) | 121 (96.8%) | 63^A^ (52.1%) | 6 (31.3%) | 41 (53.2%) | 3 (42.9%) | 13 (61.9%) | 4.58 (3), p=.209 |  |
| Other seizure types, n (%) | 121 (96.8%) | 106 (87.6%) | 16 (84.2%) | 66 (89.2%) | 7 (100.0%) | 17 (77.2%) | 2.22 (3), p=.557 |  |
| Age of seizure onset in months, mean (SD) [range] | 109 (87.2%) | 11.96 (17.96) [0-122] | 30.88 (32.69) [3-122] | 9.05 (10.55) [0-60] | 14.43 (25.51) [1-72] | 5.18 (4.06) [0.75-18.00] | 9.07 (3,105), p<.001 | TSC2 / NMI < TSC1 |
| Seizure severity year 1, mean (SD) [range] | 125 (100.0%) | 0.00 (0.98) [-1.16–1.58] | -0.70 (0.85) [-1.16–2.43] | 0.09 (1.00) [-1.16–1.58] | 0.10 (0.98) [-1.16–1.28] | 0.25 (0.74) [-1.16–1.27] | 4.25 (3,121), p=.007 | TSC1 < TSC2 / no testing |
| Seizure severity year 2, mean (SD) [range] | 125 (100.0%) | 0.00 (0.98) [-1.30–1.22] | -0.41 (1.08) [-1.30–1.22] | 0.04 (0.98) [-1.30–1.22] | 0.11 (0.78) [-1.30–1.07] | 0.19 (0.89) [-1.30–2.51] | 1.49 (3,121), p=.222 |  |
| Epileptic spasm severity year 1, mean (SD) [range] | 120 (96.0%) | 0.00 (0.98) [-0.82–1.91] | -0.63 (0.57) [-0.82–1.11] | 0.09 (1.01) [-0.82–1.91] | -0.09 (1.18) [-0.82–1.19] | 0.28 (0.93) [-0.82–1.46] | 3.67 (3,116), p=.014 | TSC1 < TSC2 / no testing |
| Epileptic spasm severity year 2, mean (SD) [range] | 120 (96.0%) | 0.00 (0.98) [-0.59–2.07] | -0.28 (0.77) [-0.59–1.90] | 0.05 (1.03) [-0.59–2.07] | -0.14 (1.08) [-0.59–2.07] | 0.10 (1.03) [-0.59– 1.98] | 0.67 (3,116), p=.574 |  |
| Other seizures severity year 1, mean (SD) [range] | 119 (95.2%) | 0.00 (0.98) [-0.84–2.00] | -0.36 (0.84) [-0.84–1.46] | 0.03 (0.99) [-0.84–2.00] | 0.73 (0.88) [-0.84–2.67] | -0.06 (0.99) [-0.84–1.57] | 2.22 (3,115), p=.089 | TSC1 < NMI |
| Other seizures severity year 2, mean (SD) [range] | 119 (95.2%) | 0.00 (0.97) [-1.19–1.46] | -0.45 (0.96) [-1.19–1.21] | 0.06 (0.98) [-1.19–1.46] | 0.29 (0.83) [-1.19–1.20] | 0.07 (0.89) [-1.19–1.21] | 1.69 (3,115), p=.172 |  |
| Seizure severity age 7+ years, mean (SD) [range] | 94 (75.2%) | 0.00 (0.98) [-1.19–1.79] | 0.17 (1.11) [-1.19–1.79] | 0.01 (0.98) [-1.19–1.70] | 0.04 (1.04) [-1.19–1.39] | -0.23 (0.87) [-1.19–0.90] | 0.40 (3,90), p=.752 |  |
| ASD |  |  |  |  |  |  |  |  |
| ADI-R diagnostic algorithm score, mean (SD) [range] | 85 (68.0%) | 30.40 (16.28) [1–60] | 27.93 (16.53) [9-54] | 30.00 (15.51) [1-60] | 34.04 (22.51) [5-55] | 33.49 (18.31) [5-57] | 0.35 (3,81), p=.788 |  |
| ADI-R current behaviour algorithm score, mean (SD) [range] | 85 (68.0%) | 20.54 (14.90) [0–55] | 18.22 (12.76) [5-42] | 19.39 (14.20) [0-48] | 25.32 (21.86) [2-49] | 25.93 (17.54) [3-55] | 0.97 (3,81), p=.410 |  |
| ADOS-2 Comparison Score^B^, mean (SD) [range] | 80 (64.0%) | 4.53 (3.01) [0-10] | 3.98 (2.81) [0-9] | 4.42 (3.00) [0-10] | 4.44 (3.60) [0-7] | 5.59 (3.22) [0-10] | 0.72 (3,76), p=.542 |  |
| ASD factor score, mean (SD) [range] | 86 (68.8%) | 0.00 (0.97) [-1.60-2.01] | -0.17 (0.87) [-1.16-1.42] | -0.05 (0.93) [-1.60-1.67] | 0.28 (1.42) [-1.44-1.73] | 0.30 (1.11) [-1.44-2.01] | 0.76 (3,82), p=.523 |  |
| ASD research classification | 86 (68.8%) |  |  |  |  |  |  |  |
| No ASD |  | 16 (18.6%) | 3 (18.8%) | 10 (19.2%) | 1 (25.0%) | 2 (14.3%) | 4.43 (9), p=.961 |  |
| Broad spectrum |  | 11 (12.8%) | 3 (18.8%) | 7 (13.5%) | 0 (0.0%) | 1 (7.1%) |  |  |
| Probable ASD |  | 25 (29.1%) | 6 (37.5%) | 14 (26.9%) | 1 (25.0%) | 4 (28.6%) |  |  |
| ASD |  | 34 (39.5%) | 4 (25.0%) | 21 (40.4%) | 2 (50.0%) | 7 (50.0%) |  |  |
| Estimated IQ, mean (SD) [range] | 124 (99.2%) | 69.57 (20.80) [29-119] | 77.68 (23.33) [38-119] | 68.21 (19.00) [29-112] | 66.57 (23.31) [42-101] | 68.23 (23.50) [40-118] | 1.16 (3,120), p=.330 |  |

# **Supplementary Section 2: Tuber count**

Copies of clinical brain scans were obtained from the hospitals where imaging had been conducted during Phase 1, when possible (n=86). During Phase 2, more recent clinical scans were obtained for 41 participants and new scans for 23 participants were obtained (total n=109). Total tuber count ranged from 0-68 (Figure S1, panel A).

**A	B**

**C**	**D**

**E	F**

### **Figure S1. Tuber load in whole brain (A) and each lobe separately (B-F)**

**A	B**

**Figure S2. Tuber load in whole brain shown by mutation status, for entire sample with tuber data (A; N=109) and for subsample with both Phase 2 autism assessment and tuber data (B; N=80)**
TSC1, mutation identified in TSC1 gene; TSC2, mutation identified in TSC2 gene; NMI, no mutation identified

### **Table S2. Cortical tuber count by major lobe and genetic mutation status**

|  | *Tuber count, mean (SD) [range]* | | | | |  |  |  |
| --- | --- | --- | --- | --- | --- | --- | --- | --- |
|  | *Overall* | *TSC1 mutation (n=19)* | *TSC2 mutation (n=77)* | *No mutation identified (n=7)* | *Not tested (n=22)* | *F(df), p* |  | *Posthoc tests* |
| Whole brain | 19.31 (14.42)  [0-68] | 6.13 (6.40)  [0-22] | 22.01 (14.54) [0-68] | 27.86 (14.92)  [7-48] | 16.89 (12.16)  [0-43] | 6.96 (3,105), p<.001 |  | TSC1 < TSC2/NMI/no testing |
| Frontal lobes | 10.07 (7.45)  [0-31] | 3.80 (4.30) [0-14] | 11.41 (7.31)  [0-31] | 13.57 (8.24)  [3-26] | 8.95 (7.17) [0-24] | 5.56 (3,105), p=.001 |  | TSC1 < TSC2/NMI/no testing |
| Temporal lobes | 2.94 (3.02)  [0-16] | 0.87 (1.19) [0-3] | 3.37 (3.19)  [0-16] | 4.14 (4.06)  [0-12] | 2.63 (2.31)  [0-7] | 3.48 (3,105), p=.019 |  | TSC1 < TSC2/no testing |
| Parietal lobes | 3.24 (2.55)  [0-11] | 1.00 (1.51)  [0-5] | 3.71 (2.68)  [0-11] | 3.86 (2.12)  [1-8] | 3.11 (1.94)  [0-6] | 5.37 (3,105), p=.002 |  | TSC1 < TSC2/no testing |
| Occipital lobes | 2.94 (3.28)  [0-18] | 0.47 (0.64)  [0-2] | 3.38 (3.45)  [0-18] | 6.14 (3.81)  [0-11] | 2.16 (2.12)  [0-7] | 6.79 (3,105), p<.001 |  | TSC1 < TSC2/NMI/no testing |
| Cerebellar lobes | 0.11 (0.46)  [0-3] | – | 0.15 (0.55)  [0-3] | 0.14 (0.38)  [0-1] | 0.05 (0.23)  [0-1] | 0.54 (3,105), p=.656 |  |  |

Note. N=109 with brain scan data.

Figure S3 shows results of the factor analysis with tuber count from frontal, temporal, parietal, and occipital lobes loading on a latent factor representing tuber burden. Tuber count from cerebellar lobes did not load significantly onto the latent factor and so was dropped from the model; this likely reflects the small number of children with cerebellar tubers (n=8). The final model fit the data well (χ^2^(2)=1.98, p=.373; RMSEA=0.00, 90%CI 0.00-0.19; CFI=1.00; SRMR=0.018).

### **Figure S3. Confirmatory factor analysis of cortical tuber load based on most recent or best quality scan**

# **Supplementary Section 3. Epilepsy**

Of 125 participants, 114 (91%) had a history of epilepsy. A detailed seizure history was obtained from the parents using a specially devised epilepsy interview schedule that enquired about the manifestations of possible seizures (full description in ^2^). Parents were also given a seizure diary to record seizure type and frequency over a two-week period, as well as the drug regime and medication changes. Details from the parent interview were cross-checked against and supplemented with information from contemporaneous medical records and the summary information was used to determine the key features of the epilepsy. Medical notes and parent narratives were reviewed and scored by independent raters (Phase 1: PFB, MC, BN; Phase 2: HL, PFB, FO). Consensus coding was established by two of the raters reviewing the narratives. For most of the patients there were multiple sources of information for each time period. When there was a disagreement, additional information from doctors and the family was obtained to identify the most valid score. Epilepsy severity was assessed for the first year of life, second year of life, current Phase 1 (preceding 3 months) and current Phase 2 (preceding 3 months), with a range of 0-3 points for each feature included. Scores for the first year of life, second year of life, and current Phase 2 were included in analyses.

## **3.1 Epileptic spasms**

In nearly all cases (93.7%) spasms co-occurred with other seizure types. Of those with spasms, for the majority these occurred over a period of one to six months (28.6%) or greater than six months (51.8%). For 19.6% they occurred for a duration of less than one month. The vast majority (87.5%) had spasms more than once daily at the period of greatest frequency. In 68.3% of cases one or two drugs were used and in 20.6% of cases more than two drugs were required (no drugs were given or data were missing in the remaining cases). Of those in which treatment was given, in 78.2% of cases there was a complete response to drugs, in 10.9% there was a partial response (50% reduction) and in 10.9% there was poor response (less than 50% reduction). Vigabatrin was used in 71.4% at the time of spasms, resulting in complete cessation in 77.8% and partial response in 13.3% of cases. Best response was achieved almost immediately (i.e., within 1-2 days) in 67.5% of cases. A quarter (24.4%) of these cases were additionally treated with steroids and a small proportion of those with spasms were treated with steroids and not vigabatrin (8.2%).

## **3.2 Other seizure types**

One hundred participants had seizure types other than spasms. More than half (54.0%) had three or more other seizure types, 27.0% had two other seizure types, and 26.0% had only one other seizure type. For the majority these occurred over a period greater than six months (86.0%). For 8.0% they occurred over one to six months, and for 6.0% for a duration of less than one month. The majority (60.5%) had seizures more than once daily at greatest frequency, although 12.8% had seizures weekly or daily and 26.7% had seizures only monthly or less frequently. In 34.0% of cases one or two drugs were used, in 44% three to five drugs were used, and in 22.0% more than five were required. Of those in which treatment was given and data about response were available, in 50.9% of cases there was a complete response, in 41.8% there was a partial response and in 7.3% there was poor response.

## **3.3 Seizure history at Phase 2 assessment (current seizures)**

A seizure history covering the three months preceding Phase 2 assessment was taken for 78 participants with ASD data and an additional 15 who did not complete ASD assessments. Around half (52.7%) had had seizures. In most cases (65.3% of those having seizures) only one seizure type had occurred, in 20.4% there were two types, and in 14.3% there were three or more. In around a third (32.3%) of those having seizures they occurred more than once daily, while 14.6% had them daily and the remainder (54.2%) had them weekly or less. For the vast majority (95.9%) the seizures had continued for more than 6 months; it was rare for current seizures to have occurred for less than 6 months (4.1%). At Phase 2 assessment 62.8% of participants were taking 1-2 epilepsy drugs and 9.6% were taking greater than 2 drugs. The response was complete in 29.2% of those taking drugs, partial in 61.5%, and there was no response in 9.2%.

## **3.4 Factor analysis of seizure data**

Exploratory factor analysis was conducted for each of the time periods to ascertain the loadings of each E-Chess feature of epilepsy to severity scores. These indicated that history of status epilepticus (none, 30 minutes to 1 hour, 1 hour or more) did not significantly load on the seizure severity factor for any time point. Status epilepticus was therefore removed from further factor analysis and not included in structural equation modelling analysis. Given the potential association between ASD likelihood and status epilepticus, separate analysis was performed to explore the association between history of status epilepticus and ASD factor scores, described in the main manuscript and below.

## **3.5 Factor analysis of seizure severity in the first two years of life**

The factor structure of seizure variables from the first two years of life was explored through EFA. Variables included were: (i) number of seizure types (separate variables for Year 1 and Year 2); (ii) time period over which seizures occur (duration) (separate variables for Year 1 and Year 2); (iii) seizure frequency at most severe (separate variables for Year 1 and Year 2); (iv) number of anti-epileptic drugs used (separate variables for Year 1 and Year 2); and (v) response to treatment (separate variables for Year 1 and Year 2). Results are shown in Table S2.

### **Table S3. Exploratory factor analysis of seizure severity in the first two years of life**

|  | Chi-square (df) | p | RMSEA | CFI | SRMR | AIC | SABIC | Comparison with previous model |
| --- | --- | --- | --- | --- | --- | --- | --- | --- |
| 1 factor | 485.50 (35) | <.0001 | 0.321 | 0.638 | 0.124 | 3433.78 | 3423.77 |  |
| 2 factors | 202.87 (26) | <.0001 | 0.233 | 0.858 | 0.052 | 3169.16 | 3156.14 | <.0001 |

Note. All seizure types are combined in ratings of each seizure feature, which includes N seizure types, duration, frequency, N antiepileptic drugs, and response to treatment separately for year 1 and year 2.

While neither model fits that well, the 2 factor solution (Factor 1=Year 1 severity, Factor 2=Year 2 severity) is preferable to the 1 factor solution. Confirmatory factor analysis was used to generate factor scores for these two variables, as shown in Figure S4.

**Figure S4. Confirmatory factor analysis of seizure severity in the first two years of life**
Factor loadings for two factors indicated by EFA: (1) Seizure severity in year 1; (2) Seizure severity in year 2.

To disentangle the effects of epileptic spasms from other seizure types in early development, the data was recoded so that the indicators shown in Figure S4 were coded separately for spasms and for all other seizure types. Further exploratory factor analysis was then carried out, using the following variables: (i) age of seizure onset, (ii) those measuring spasm severity in the first year of life, namely duration, frequency at most severe, number of anti-epileptic drugs used, and response to treatment; (iii) those measuring spasm severity in the second year of life, namely duration, frequency, number of drugs, and response to treatment; (iv) those measuring severity of other (non-spasm) seizures in the first year of life, namely number of other seizure types, duration, frequency, number of drugs, and response to treatment; and (v) those measuring severity of other (non-spasm) seizures in the second year of life, namely number of other seizure types, duration, frequency, number of drugs, and response to treatment. Results are shown in Table S3.

### **Table S4. Exploratory factor analysis of spasm and other seizure types severity in the first two years of life**

|  | Chi-square (df) | p | RMSEA | CFI | SRMR | AIC | SABIC | Comparison with previous model |
| --- | --- | --- | --- | --- | --- | --- | --- | --- |
| 1 factor | 1674.63 (152) | <.0001 | 0.288 | 0.356 | 0.242 | 5729.02 | 5708.16 |  |
| 2 factors | 978.94 (134) | <.0001 | 0.228 | 0.643 | 0.118 | 5069.34 | 5041.89 | <.0001 |
| 3 factors | 460.11 (117) | <.0001 | 0.156 | 0.855 | 0.078 | 4584.50 | 4550.84 | <.0001 |
| 4 factors | 244.40 (101) | <.0001 | 0.108 | 0.939 | 0.027 | 4400.79 | 4361.28 | <.0001 |

The 4 factor solution fit the data best, and resulted in the following factors: (1) Spasm severity in the first year of life (frequency, duration, N drugs, response to treatment, age of onset); (2) Spasm severity in the second year of life (frequency, duration, N drugs, response to treatment); (3) Other seizure severity in the first year of life (N seizure types, frequency, duration, N drugs, response to treatment, age of onset); and (4) Other seizure severity in the second year of life (N seizure types, frequency, duration, N drugs, response to treatment). Confirmatory factor analysis was used to generate factor scores for these four factors. Age of onset loaded approx. equally on both spasm and other seizure severity in year 1; given the interest in exploring the role of age of onset independently of seizure severity, it was not included in the saved factor scores. The resulting factor loadings are shown in Figure S5.

**Figure S5. Confirmatory factor analysis of epileptic spasms and other seizures in the first two years of life**
Factor loadings for four factors indicated by EFA: (1) Spasm severity in year 1; (2) Spasm severity in year 2; (3) Other seizure types severity in year 1; (4) Other seizure types severity in year 2.

## **3.6 Factor analysis of current seizure severity**

Finally, confirmatory factor analysis was used to generate a factor score for seizure severity in the 3 months preceding Phase 2 assessment (age 7+ years), as shown in Figure S. While this model did not show particularly good fit to the data (χ^2^ (5) = 49.84, p<.0001; RMSEA = 0.309, 90%CI 0.234-0.390; CFI = 0.854; SRMR = 0.048), the factor loadings were significant and consistent with results from the first two years of life.

### **Figure S6. Confirmatory factor analysis of seizure severity at 7+ years**

## **3.7 Seizure severity and onset by genetic status**

Seizure severity differed by genetic status for seizures in the first year of life (F(df)= 4.25 (3,121), p=.007) and for epileptic spasms in the first year of life (F(df)=3.67 (3,116), p=.014); boxplots are shown in Figure S7.

**A	B**

**Figure S7. Boxplots showing distribution of (A) seizure severity factor score in year 1 and (B) epileptic spasms severity factor score in year 1 by mutation status**
NMI, no mutation identified; TSC1, mutation identified in TSC1 gene; TSC2, mutation identified in TSC2 gene.

**Figure S8. Boxplot showing distribution of age of seizure onset in months by mutation status**
NMI, no mutation identified; TSC1, mutation identified in TSC1 gene; TSC2, mutation identified in TSC2 gene. Distributions are significantly different (Kruskal-Wallis Test [df] = 14.20 [3], p=.003).

# **Supplementary Section 4. Autism likelihood**

Assessment for autism spectrum disorder was carried out in N=86 individuals: in 79 cases both ADI-R^3^ and ADOS-2^4^ data were available, in 6 cases only ADI-R data were available, and in 1 case only ADOS-2 data were available. Fourteen individuals (17.5%) completed ADOS-2 Module 1, 7 (8.8%) completed Module 2, 40 (50.0%) completed Module 3, and 19 (23.8%) completed module 4.

## **4.1 ADI-R algorithms**

Modified ADI-R AGRE criteria^5^ were used to classify children as having autism, not quite autism (ASD), broad spectrum, or below all thresholds. Algorithms were based on those developed by the Autism Genetic Resource Exchange (AGRE) with slight modification to the broad spectrum classification, and are as follows (a*ll of the following algorithms are based on ADI-R domain scores):*

I. "AUTISM" classification:
Uses the validated ADI-R diagnostic algorithm:^6^

1. social >= 10, and
2. communication: verbal >= 8; nonverbal >= 7, and
3. behaviour >= 3
PLUS age of onset >= 1

II. "NOT QUITE AUTISM (NQA)" classification:

EITHER:

(A) Meets cut-offs on all 3 "content" domains, but not age of onset domain:
1. social >= 10, and
2. communication: verbal >= 8; nonverbal >= 7, and
3. behaviour >= 3
PLUS age of onset = 0

OR:

(B) Is no more than 1 point below cut-off on any, or all, of the 3 "content" domains, and meets “age of onset” domain:
1. social >= 9, and
2. communication: verbal >= 7; nonverbal >= 6, and
3. behaviour >= 2
PLUS age of onset >=1

III. "BROAD SPECTRUM" classification:

Age of onset >= 0;
PLUS does not meet criteria for Autism or NQA;
PLUS meets one or more of the following (A or B):

(A) Shows severe deficit on at least two domains; severe is defined by scores at two or more of the following levels (i.e., 1 + 2, or 2 + 3, or 1 + 3):
1. social >= 8, and/or
2. communication: verbal >= 7; nonverbal >= 6, and/or
3. behaviour >= 3

(B) Shows more moderate deficits, but in all three domains at the following levels; moderate is defined by scores at all of the following levels:
1. social >= 4, and
2. communication >= 3 (nonverbal or verbal), and
3. behaviour >= 2

## **4.2 ADOS-2 algorithms**

ADOS-2 criteria classified children as meeting criteria for autism, ASD, or being below cut-off using the following cut-offs:

I. “AUTISM” classification:

Module 1, Few To No Words
Overall Total score ≥16

Module 1, Some Words
Overall Total score ≥12

Module 2, Younger Than 5 Years^[1]^
Overall Total score ≥10

Module 2, Aged 5 Years Or Older
Overall Total score ≥9

Module 3
Overall Total score ≥9

Module 4
1. Communication and Social Interaction score ≥10, and
2. Communication score ≥3, and
3. Social Interaction score ≥6

II. “AUTISM SPECTRUM” classification:

Does not meet criteria for AUTISM, and:

Module 1, Few To No Words
Overall Total score ≥11

Module 1, Some Words
Overall Total score ≥8

Module 2, Younger Than 5 Years^1^
Overall Total score ≥7

Module 2, Aged 5 Years Or Older
Overall Total score ≥8

Module 3
Overall Total score ≥7

Module 4
1. Communication and Social Interaction score ≥7, and
2. Communication score ≥2, and
3. Social Interaction score ≥4

## **4.3 ASD research diagnosis**

Sixteen children (18.6%) were below all cut-offs on both ADI-R and ADOS-2 and were classified as *No ASD*. Thirty-four children (39.5%) met both ADI-R and ADOS-2 criteria for ASD or autism and were classified as *ASD*. Twenty-five children (29.1%) met either ADI-R or ADOS-2 criteria and were classified as *Probable ASD*. Finally, 11 children (12.8%) met ADI-R AGRE criteria for broad spectrum and did not meet ADOS criteria, so were classified as *Broad Spectrum*.

## **4.4 ASD factor analysis**

Mean score for the ADI-R diagnostic algorithm was 30.40 (SD=16.28; median=29; range=1-60) and for the ADI-R current behaviour algorithm was 20.54 (SD=14.90; median=17; range=0-55). Mean ADOS comparison score was 4.53 (SD=3.01; median=4; range=0-10). Confirmatory factor analysis was used to generate a factor score reflecting severity of autism symptoms, as shown in Figure S7. This model was saturated, with 0 degrees of freedom (χ^2^ (0) = 00.00, p=.0000). The use of the ADI-R current behaviour algorithm score and the ADOS-2 score (both measuring behaviour at age 7 years or older), alongside the ADI-R diagnostic algorithm score, reduces the risk that autism severity scores are confounded by low developmental level at the age of 4-5 years, the period that the diagnostic algorithm focuses on. ASD factor score was higher in males than females (mean [SD]=0.24 [.95] vs. -.20 [.95], t[df]=2.15 [84], p=.034, d=.46).

### **Figure S9. Confirmatory factor analysis of autism severity at 7+ years**

ASD factor scores significantly differed between ASD diagnostic groupings (F(3,82)=32.23, p<.001). Posthoc tests demonstrated significant differences between all groups: No ASD < Broad Spectrum < Probable ASD < ASD. See Figure S10.

### **Figure S10. ASD latent factor score in each diagnostic grouping**

## **4.5 ASD factor score distribution**

The ASD latent factor score appeared to have a bimodal distribution. However, this was driven by a small number of nonverbal or minimally verbal children (those who completed an ADOS-2 Module 1 or 2) and there was not strong evidence of bimodality overall (Bimodality Coefficient = .52; values > .55 suggest a bimodal distribution^7,8^). However, the relatively small sample size and small distance between the two observed peaks may have limited the ability to detect bimodality; thus true bimodality cannot be ruled out and a larger sample size will be necessary to explore this in more detail. See Figure S11.

**A**

**B	C**

### **Figure S11. ASD latent factor score in (A) all those with ASD data, (B) those who completed ADOS-2 Module 1 or 2 (nonverbal or minimally verbal), and (C) those who completed ADOS-2 Module 3 or 4 (fluent speech)**

**A	B**

**C	D**

 **E**

### **Figure S12. ASD factor score in those with (A) TSC1 mutation (B), TSC2 mutation (C), no mutation identified (D), and no genetic testing carried out**

## **4.6 Tuber location and ASD severity**

Previous research has shown an association between the presence of tubers in the temporal lobe and autism likelihood.^9^ We examined correlations between tuber count in each lobe and ASD factor score (Table S4). Tuber count in frontal and temporal lobes correlated modestly with ASD factor score, as did the total tuber count for the whole brain. Tuber counts for frontal, temporal, parietal, and occipital lobes were strongly correlated with each other and with total tuber count.

To test whether tubers in frontal and temporal lobes were associated with ASD factor score when controlling for total tuber count, we first conducted a series of multiple linear regressions with ASD factor score as the dependent variable, and tuber count from one lobe and total tuber count as independent variables. A separate model was run for each lobe (n=80). Neither frontal (β = .52, p=.162) nor temporal (β = .09, p=.653) lobe tuber count was a significant predictor of ASD factor score when controlling for total tuber count (Frontal lobe model, total tuber count β = -.26, p=.483; Temporal lobe model, total tuber count β = .17, p=.384). However, the strong correlation between tuber count for individual lobes and total tuber count resulted in a significant degree of collinearity in these models making it difficult to interpret these results.

As an alternative approach, multiple linear regressions were run with ASD factor score as the dependent variable and presence vs. absence of tubers in each lobe and total tuber count as independent variables; this avoided significant collinearity. Presence of tubers in frontal (β = -.05, p=.651) and temporal (β = .09, p=.535) lobes did not predict ASD factor score when controlling for total tuber count (Frontal lobe model, total tuber count β = .26, p=.034; Temporal lobe model, total tuber count β = .18, p=.199).

|  | **Tuber count frontal lobes** | **Tuber count temporal lobes** | **Tuber count parietal lobes** | **Tuber count occipital lobes** | **Tuber count cerebellar lobes** | **Total tuber count all lobes** | **ASD** |
| --- | --- | --- | --- | --- | --- | --- | --- |
| Tuber count frontal | 1.00 (109) |  |  |  |  |  |  |
| Tuber count temporal | .71** (109) | 1.00 (109) |  |  |  |  |  |
| Tuber count parietal | .79** (109) | .63** (109) | 1.00 (109) |  |  |  |  |
| Tuber count occipital | .68** (109) | .65** (109) | .66** (109) | 1.00 (109) |  |  |  |
| Tuber count cerebellar | .15 (109) | .12 (109) | .04 (109) | .01 (109) | 1.00 (109) |  |  |
| Total tuber count | .95** (109) | .83** (109) | .85** (109) | .81** (109) | .13 (109) | 1.00 (109) |  |
| ASD | .27* (80) | .25* (80) | .19 (80) | .15 (80) | .06 (80) | .27* (80) | 1.00 |

**Table S5. Correlations (Spearman’s rho) between tuber count and ASD factor score (N with data in parentheses).**
ASD is factor score representing severity of symptoms. ** p<.01, * p<.05

## **4.7 Seizures and ASD severity**

Those with spasms had significantly higher ASD factor scores compared to those without spasms (n=86, mean [SD] = 0.31 [0.89] vs. -0.36 [0.94], p=.001, d=0.74; see Figure S13), and those with at least one episode of status epilepticus (SE) had significantly higher ASD factor scores than those who had not had SE (n=85, mean [SD] = 0.30 [0.99] vs. -0.21 [0.90], p=.014, d=0.54; see Figure S14). Twenty-four children had neither spasms nor SE and had significantly lower ASD factor scores than those who had a history of spasms and/or SE (n=86, mean [SD] = -0.65 [0.81] vs. 0.25 [0.91], p<.001, d=1.04; see Figure S15). However, there remains suggestion of a bimodal distribution of ASD factor scores in those with or without spasms and/or SE (Figures S13-S15), suggesting that other factors are at play. History of spasms and SE were entered into regression analysis with severity of other seizure types (coded for ‘ever’, using data from all study periods) included as a covariate (n=85). Spasms (B=0.59, p=.002) and other seizure severity (B=0.05, p=.019) were both significant, with SE being of borderline significance (B=0.39, p=.051). Repeating this analysis with other seizure severity recoded into a dichotomous variable (no to low severity vs. medium to high severity) confirmed that both spasms (B=0.65, p<.001) and other seizure types (B=0.74, p<.001) played an independent role, but SE was of marginal significance (B=0.34, p=.068). Repeating this model with all possible interactions showed that there was no significant interaction between seizure types (all p>.254). These differences remained when controlling for sex; while males had significantly higher ASD factor scores (female vs. male, B=-0.51, p=.004), coefficient estimates for spasms and other seizure types were of similar magnitude. Repeating the analysis controlling for IQ showed that spasms remained significant (B=0.36, β=0.18, p=.047), whereas SE (B=.15, β=0.08, p=.398) and other seizure severity (B=0.30, β=0.15, p=.150) were not significant; IQ was significantly associated with ASD factor score (B=-0.02, β=-0.47, p<.001). Figure S16 shows different combinations of epilepsy risk factors.

**A	B**

### **Figure S13. ASD factor score in those with (A) no history of epileptic spasms and (B) epileptic spasms**

**A	B**

### **Figure S14. ASD factor score in those with (A) no history of status epilepticus and (B) at least one episode of status epilepticus**

**A	B**

### **Figure S15. ASD factor score in those with (A) no history of epileptic spasms and no status epilepticus and (B) epileptic spasms and/or at least one episode of status epilepticus**

ASD factor score

|  | Mean ASD factor score |
| --- | --- |
| No seizures | -0.93 |
| SE only | -0.75 |
| Other seizure types only | -0.41 |
| Spasms only | -0.39 |
| Spasms and SE | 0.1 |
| Other seizure types and SE | 0.26 |
| Spasms and Other seizure types | 0.58 |
| Spasms, Other seizure types, and SE | 0.61 |

**Figure S16. ASD factor score in those with differing seizure history**
No seizures, N=11; Status Epilepticus (SE) only, N=3; Other seizure types only, N=13; Epileptic spasms only, N=11; Epileptic spasms and SE, N=5; Other seizure types and SE, N=13; Epileptic spasms and Other seizure types, N=14; Epileptic spasms, Other seizure types, and SE, N=15.

**Figure S17. Estimated marginal means for ASD factor scores by epilepsy risk factors**

Estimated marginal means from ANOVA with epileptic spasms (no spasms vs. history of spasms; *F*(df)=13.44(1,81), *p*<.001), status epilepticus (no status epilepticus vs. history of status epilepticus; *F*(df)=3.42(1,81), *p*=.068), and other seizure types severity (no seizures or low severity vs. moderate to high severity; *F*(df)=15.28(1,81), *p*<.001) as between subjects factors.

# **Supplementary Section 5. Intellectual ability**

## **5.1 Phase 1**

Intellectual abilities were assessed using the Mullen Scales of Early Learning (MSEL^10^) in participants up to 68 months of age (n=55). The assessments were undertaken at or around the age of 2 years or at recruitment if the child was over 2 years of age at the time of diagnosis. Adaptive level was assessed using the Vineland Adaptive Behaviour Scales extended survey parental interview (n=113^11^). Vineland adaptive composite scores were used to estimate IQ if the child was above the recommended age for administration of the Mullen Scales (n=35). In addition, the Vineland adaptive composite score was used when administration of the Mullen Scales was not possible (n=5), or when the Mullen Scales standard score was at ‘floor’ level (n=26).

## **5.2 Phase 2**

The Wechsler Abbreviated Scale of Intelligence – Second Edition (WASI-2^12^) was administered where possible (n=57). Full-scale IQ from two sub-tests (vocabulary, matrix reasoning) was used for two participants (n=2). One participant completed the British Picture Vocabulary Scale (BPVS^13^). The Vineland Adaptive Behaviour Scales, Second Edition (VABS-II^14^) were administered to 81 participants. The adaptive behaviour composite from the VABS-II was used to estimate IQ when the participant was not able to complete the WASI-2 or the BPVS, either due to level of functioning (n=24) or when administration of the WASI-2 was not possible (n=2). All four sub-tests of the WASI-2 were administered to 35 unaffected siblings of the TSC participants.

## **5.3 Estimated IQ**

There was a moderate correlation between the WASI-2 and VABS-II scores at Phase 2 (n=54; rho=.50, p<.001), but a stronger correlation between the WASI-2 scores at Phase 2 and MSEL at Phase 1 (n=34; rho=.69, p<.001) and between MSEL and VABS at Phase 1 (n=73; rho=.72, p<.001). A best-estimate IQ variable was created by using the WASI-II where available, followed by the MSEL, VABS-II, or VABS (in that order, where a valid score was available and was not at floor level). This resulted in a best-estimate IQ for N=124 participants.

## **5.4 Estimated IQ and ASD**

Previous analyses in this sample^15,16^ has shown that severity of seizures or presence of spasms predicts IQ. It was important to determine whether the observed effect of seizure severity on ASD severity was driven by the effect on IQ, including the possibility that high scores in the ADI-R and ADOS were an artefact of very low IQ. Estimated IQ was significantly correlated with ASD factor score (rho=-.61, p<001) in the group as a whole. However, a scatterplot of ASD factor score plotted against estimated IQ showed that there was a wide range of ASD factor scores in those with IQ below 70, including individuals from all diagnostic groups (see Figure S17). Of those with the highest ASD factor scores (above 1.0), there were individuals with IQ ranging from approximately 40 to 90. Furthermore, the association between IQ and ASD factor scores was moderate and nonsignificant within groups (ASD rho=-.29, p=.092, n=34; Probable ASD rho=-.49, p=.014, n=25; Broad spectrum rho = -.44, p=.172, n=11; No ASD rho=-.04, p=.890, n=16). This suggests that while the likelihood for ASD and low IQ are linked, there is also considerable variation in the way that this presents (e.g., high likelihood of ASD but IQ in the normal range, or very likelihood risk of ASD but IQ in the range of mild to moderate intellectual impairment).

**Figure S18. Scatterplot of ASD factor score by estimated IQ for each diagnostic group**
*No ASD*, R^2^=.00; *Broad spectrum*, R^2^=.33; *Probable ASD*, R^2^=.20; *ASD*, R^2^=.17.

# **Supplementary Section 6. Bivariate associations**

Genetic status was not associated with ASD factor score or ASD diagnostic groupings (see Table 1, main manuscript). Those with TSC1 mutation (versus all other groups) had significantly lower cortical tuber count (see Tables 1 and S1), with a large difference between TSC1 and TSC2 (6.13 vs. 22.01, d=1.41). While spasms were more common in TSC2 vs. TSC1 mutation (53.2% vs. 31.3%), there was no significant association between spasms and genetic status overall (χ2[df]=4.58[3], p=.209). Those with TSC1 had lower seizure severity scores in the first year of life versus TSC2 and those with no genetic testing, and higher age of seizure onset (TSC1 median=20 months vs. 5-6 months [TSC2 mutation/no mutation identified; NMI]). See Table 1 and Figures S7 and S8.

Correlations between all variables are presented in Table S5. Mutation (TSC2 vs. TSC1; excluding those with no testing and NMI) was associated with higher tuber count, lower age of seizure onset, spasms in the first and second years of life, and other seizure types in the second year of life. Mutation was not associated with any of the variables at Phase 2 assessment. Tuber count was associated with all early seizure variables, ASD, and estimated IQ. Spasms severity in the first two years of life was associated with other seizure types in the second year, though other seizure types in the first year of life were not associated with spasms. This may reflect progression from spasms to other seizure types in infancy. Severity of other seizure types in the second, and to a lesser extent the first, year of life was associated with seizures at 7+ years, suggesting moderate stability of seizures into at least middle childhood. Severity of all seizure types in infancy and at Phase 2 was associated with ASD symptoms. Estimated IQ was associated with ASD factor score and with all risk factors.

|  | **Mutation, TSC1 vs. TSC2^A^** | **Cortical tuber count** | **Age at seizure onset** | **Spasms year 1** | **Spasms year 2** | **Other seizures year 1** | **Other seizures year 2** | **Seizures age 7+ years** | **Estimated IQ** | **ASD factor score** |
| --- | --- | --- | --- | --- | --- | --- | --- | --- | --- | --- |
| Mutation, TSC1 vs. TSC2^A^ | 1.00 (96) |  |  |  |  |  |  |  |  |  |
| Cortical tuber count | .45** (83) | 1.00 (109) |  |  |  |  |  |  |  |  |
| Age at seizure onset | -.37** (84) | -.33** (97) | 1.00 (109) |  |  |  |  |  |  |  |
| Spasms  year 1 | .30** (93) | .42** (104) | -.45** (108) | 1.00 (120) |  |  |  |  |  |  |
| Spasms  year 2 | .22* (93) | .31** (104) | -.36** (108) | .75** (120) | 1.00 (120) |  |  |  |  |  |
| Other seizures year 1 | .15 (92) | .20* (104) | -.67** (107) | .17 (118) | .13 (118) | 1.00 (119) |  |  |  |  |
| Other seizures year 2 | .21* (92) | .33** (104) | -.46** (107) | .28** (118) | .33** (118) | .61^**^ (119) | 1.00 (119) |  |  |  |
| Seizures age 7+ years | -.06 (74) | .06 (88) | -.13 (88) | .07 (92) | .08 (92) | .28^**^ (91) | .51^**^ (91) | 1.00 (94) |  |  |
| Estimated IQ | -.16 (95) | -.27** (109) | .35** (109) | -.35** (119) | -.40** (119) | -.40** (118) | -.53** (118) | -.45** (94) | 1.00 (124) |  |
| ASD factor score | .07 (68) | .27* (80) | -.20 (79) | .34** (85) | .32** (85) | .24* (84) | .30** (84) | .32** (80) | -.61** (86) | 1.00 (86) |

**Table S6. Correlations (Spearman’s rho) between ASD and all risk variables (N with data in parentheses).**
Spasms, other seizures and ASD are all factor scores representing severity of symptoms. ^A^ Only those with TSC1 or TSC2 mutation included for correlations including genetic mutation status; those with no mutation identified or not tested were excluded. ** p<.01, * p<.05

# **Supplementary Section 7. Structural Equation Models (SEM) tested**

| Model | | N sample statistics | Chi-square (df) | p | RMSEA^A^ (90% CI) | CFI | SRMR^B^ | AIC^C^ | BIC^C^ | SABIC^C^ |
| --- | --- | --- | --- | --- | --- | --- | --- | --- | --- | --- |
| 1a | Full mediation model with spasms and other seizure types separately for year 1 and 2 | 66 | 28.68 (18) | .052 | 0.069 (0.000-0.114) | 0.974 | 0.051 | 4585.11 | 4751.98 | 4565.41 |
| 1b | Mediation model with year 1+2 latent factors for spasms and other seizures | 66 | 37.43 (27) | .087 | 0.056  (0.000-0.095) | 0.974 | 0.050 | 4574.51 | 4715.92 | 4557.81 |
| 2 | Mediation model as 1b but adding age of seizure onset | 78 | 46.15 (31) | .039 | 0.063 (0.014-0.098) | 0.967 | 0.043 | 5467.47 | 5634.34 | 5447.77 |
| 3a | Mediation model with all seizure types together but separately for year 1 and 2 | 45 | 23.07 (12) | .027 | 0.086 (0.028-0.138) | 0.970 | 0.035 | 3947.85 | 4066.64 | 3933.83 |
| 3b | Mediation model with year 1+2 latent factor for all seizure types | 45 | 36.47 (16) | .003 | 0.101 (0.058-0.145) | 0.944 | 0.049 | 3951.60 | 4059.07 | 3938.91 |

### **Table S7: Fit statistics for Structural Equation Models**

AIC = Akaike information criterion; BIC = Bayesian information criterion; CFI = comparative fit index; RMSEA = root mean square error of approximation; SABIC = sample-size-adjusted BIC; SRMR = standardized root mean squared residual.

^A^ The lower value of the 90% confidence interval should include or be very near zero (or no worse than 0.05) and the upper value should not be very large, i.e., less than .08; RMSEA penalises greater complexity.

^B^ SRMR does not penalise for model complexity.

^C^ AIC and BIC are comparative fit indices, lower values indicate better fitting models; penalise complexity; can only be used to compare nested models with same observed variables.

**1a. Full mediation model with spasms and other seizure types separately for year 1 and 2**

**1b. Mediation model with year 1+2 latent factors for spasms and other seizures**

**2. Mediation model as 1b but adding age of seizure onset**

**3a. Mediation model with all seizure types together**

**3b. Mediation model with year 1+2 latent factor score for all seizure types**

### **Figure S19. SEM models tested**

# **References**

1.	Roach ES, DiMario FJ, Kandt RS, Northrup H. Tuberous Sclerosis Consensus Conference: recommendations for diagnostic evaluation. National Tuberous Sclerosis Association. *J Child Neurol.* 1999;14(6):401-407.

2.	Humphrey A, MacLean C, Ploubidis GB, et al. Intellectual development before and after the onset of infantile spasms: a controlled prospective longitudinal study in tuberous sclerosis. *Epilepsia.* 2014;55(1):108-116.

3.	Lord C, Rutter M, Le Couteur A. Autism Diagnostic Interview-Revised: a revised version of a diagnostic interview for caregivers of individuals with possible pervasive developmental disorders. *J Autism Dev Disord.* 1994;24(5):659-685.

4.	Lord C, Rutter M, DiLavore P, Risi S, Gotham K, Bishop S. *Autism Diagnostic Observation Schedule Second Edition (ADOS-2) Manual (Part 1): Modules 1–4.* Torrance, CA: Western Psychological Services; 2012.

5.	AGRE. Autism Genetic Resource Exchange. 2014; http://research.agre.org/program/diag.cfm. Accessed 6 June, 2014.

6.	Rutter M, Le Couteur A, Lord C. *ADI-R Autism Diagnostic Interview-Revised.* Los Angeles, CA: Western Psychological Services; 2008.

7.	Pfister R, Schwarz KA, Janczyk M, Dale R, Freeman JB. Good things peak in pairs: a note on the bimodality coefficient. *Front Psychol.* 2013;4:700.

8.	Freeman JB, Dale R. Assessing bimodality to detect the presence of a dual cognitive process. *Behav Res Methods.* 2013;45(1):83-97.

9.	Bolton PF, Park RJ, Higgins JN, Griffiths PD, Pickles A. Neuro-epileptic determinants of autism spectrum disorders in tuberous sclerosis complex. *Brain.* 2002;125(Pt 6):1247-1255.

10.	Mullen E. *Mullen Scales of Early Learning.* Circle Pines, MN: American Guidance Service; 1995.

11.	Sparrow SS, Balla DA, Cicchetti DV, Harrison PL. Vineland adaptive behavior scales:  Interview edition, survey form. In. Circle Pines, MN: American Guidance Service; 1984.

12.	D W. *Wechsler Abbreviated Scale of Intelligence – Second Edition (WASI-II).* San Antonio, TX: Psychological Corporation; 2011.

13.	Dunn LM, Whetton C, Dunn LM. British Picture Vocabulary  Scale  In. Windsor, UK: NFER Nelson; 1982.

14.	Sparrow SS, Cicchetti DV, Balla DA. *Vineland-II : Vineland adaptive behavior scales : survey forms manual.* Bloomington, MN: Pearson; 2005.

15.	Tye C, McEwen FS, Liang H, et al. Long-term cognitive outcomes in tuberous sclerosis complex. *Dev Med Child Neurol.* 2019.

16.	Bolton PF, Clifford M, Tye C, et al. Intellectual abilities in tuberous sclerosis complex: risk factors and correlates from the Tuberous Sclerosis 2000 Study. *Psychol Med.* 2015;45(11):2321-2331.

1. No children were under 5 years of age at ADOS-2 assessment
